# Evaluation of the Effect of Educational Courses Containing Food Safety Instructions on the Level of Knowledge, Attitude, and Practice of Pregnant Women: A Cross‐Sectional Study

**DOI:** 10.1002/hsr2.71337

**Published:** 2025-10-13

**Authors:** Fateme Asadi Touranlou, Mohammad Hashemi, Mahsa Neghabi, Shiva Adibi, Asma Afshari

**Affiliations:** ^1^ Medical Toxicology Research Center, Mashhad University of Medical Sciences Mashhad Iran; ^2^ Department of Nutrition, Faculty of Medicine Mashhad University of Medical Sciences Mashhad Iran

**Keywords:** attitude, food safety education, knowledge, pregnant women

## Abstract

**Background and Aims:**

Food safety during pregnancy significantly affects maternal health, fetal growth and development, birth outcomes, and future generations. Therefore, assessing the knowledge and attitudes of pregnant women during this period is crucial. This study aimed to examine the effects of educational food safety courses on the knowledge, attitudes, and practices of pregnant women.

**Methods:**

A cross‐sectional descriptive study was conducted from February to November 2023. Four hundred pregnant women, who were covered by healthcare centers in Mashhad, completed the questionnaire in the first phase. From this group, 144 individuals with lower levels of knowledge, attitudes, and practices were selected for the second phase. Two months after implementing the educational program, the same questionnaire was administered again to assess changes in knowledge, attitudes, and practices, which were statistically tested.

**Results:**

The majority of participants were housewives (81.3%) aged between 16 and 31 (58.8%), with a college education (68.8%), and most had lived in a metropolis (82.3%). For 30% of the respondents, this was their first pregnancy, and 57.1% were in their third trimester. Among the demographic factors analyzed, only knowledge and education levels showed a significant relationship (*p* < 0.05). Pregnant women in the intervention group demonstrated a significant increase in knowledge, attitudes, and practices regarding food safety following the educational program (*p* < 0.001).

**Conclusion:**

Increasing the knowledge, attitudes, and practices of pregnant women regarding food safety through educational pamphlets is a low‐cost and effective intervention. This approach can be expanded to a broader population through improved collaboration among government agencies and maternal and child health service providers.

## 1 Introduction

Food safety involves taking precautions to eliminate any physical, biological, or harmful agents from food that is being prepared for consumption [1, 2]. Studies indicate that annually, a significant number of people in Europe, England, Australia, and America experience foodborne illnesses, with estimates of 130 million, 2.1–3.5 million, 4.7 million, and 48 million cases respectively [3]. The majority of these illnesses are attributed to mistakes occurring during food preparation at home [2]. These findings highlight the significance of raising consumer awareness to decrease cases of foodborne illnesses.

Foodborne illnesses, considered emergencies caused by unidentified pathogens and antibiotic‐resistant pathogens, have emerged as a significant concern due to their potential to lead to severe outcomes, particularly among vulnerable populations such as pregnant women [4, 5]. Foodborne illnesses can have severe implications during pregnancy, posing risks to both the mother and the developing fetus [2, 6]. Pathogens such as Listeria, hepatitis E virus, *Toxoplasmosis gondii*, and *Coxiella burnetii* can penetrate the maternal placental unit, leading to significant health issues for both the pregnant woman and the fetus [7, 8]. Symptoms such as nausea, vomiting, diarrhea, abortion, stillbirth, and newborn blindness resulting from these pathogens may have a delayed onset [6, 8]. The most effective approach to mitigating these symptoms is through the proper preparation, storage, and hygienic handling of food [9].

The nutritional status of women during pregnancy can significantly impact women's health, fetal growth and development, birth outcomes, and the health of future generations [2, 8]. Pregnant women have a strong desire to acquire knowledge about the health aspects of nutrition during pregnancy and actively seek health information, including nutrition‐related information [10]. Therefore, it is of great importance to assess their knowledge and attitudes during this period to effectively plan for dietary habit changes. Therefore, evaluating their knowledge and attitudes during this period is crucial for planning changes in dietary habits. Studies assessing pregnant women's knowledge and attitudes of food safety in handling and examining the performance are still not positive. In a multi‐situation survey of pregnant women in the United States of America, only 18% of participants had the necessary information about foodborne pathogens [8]. The World Health Organization's recommendation in 2011 to enhance awareness, attitudes, and behaviors of pregnant women towards foodborne illnesses is crucial. Implementing interventions such as educational programs with a theoretical framework, is essential for preventing and reducing the rate of fetal loss [7, 8]. Designing and appropriately using effective educational materials that are explicit, clear, and evidence‐based to enhance the process and outcomes of healthcare is crucial [11]. Considering the absence of a comprehensive educational program on food safety for pregnant women as an effective solution for improving information dissemination to them, this study designed a food safety education program that is simple and easy to understand for participants. The study investigated the impact of this program on the knowledge, attitudes, and practices of pregnant women.

## 2 Methods

### 2.1 Study Area and Period

This cross‐sectional descriptive study was conducted from February to November 2023 in Mashhad City, Iran. The target population consisted of pregnant women who were covered by health treatment network centers in the region.

### 2.2 Inclusion and Exclusion Criteria

Inclusion criteria for the study included healthy pregnant women who provided informed consent, had completed at least elementary school, and had no complications during pregnancy. Exclusion criteria included unwillingness to participate in the educational intervention involving the review of food safety pamphlets and the occurrence of adverse events during the study period.

### 2.3 Design and Participants

This study was conducted in two phases. In the initial phase, the questionnaire adapted from Guneri et al. [2] was translated, validated, administered to 400 pregnant women receiving care at healthcare centers in Mashhad. Participants could complete the questionnaire either over the phone or in person. The questionnaire's validity and reliability were assessed as follows: A panel of experts evaluated content validity using a 4‐point Likert scale to assess the simplicity, clarity, and specificity of each question. The content validity index (CVI) was calculated for each item, and items with a CVI value less than 0.79 were excluded based on the Lawshe table (*p* > 0.05). The content validity ratio (CVR) was also assessed to evaluate the necessity of each question [12].

Content validity index = the total number of experts agreeing with the statement for 3 and 4/the total number of specialists.

To evaluate reliability and internal consistency, the Cronbach's alpha coefficient was calculated, resulting in a value of 0.75 for direct measurement constructs. For assessing stability, a test‐retest method was employed with a 10‐day interval for 10 samples, yielding a Spearman‐Brown correlation coefficient of *p* = 0.012 and *r* = 0.75. The final questionnaire consisted of four sections: demographic information (13 questions), knowledge about food safety (34 questions), attitudes towards food safety (30 questions), and practices (30 questions).

### 2.4 Intervention

In the second phase, based on research by Haghi et al. [13], we identified 144 women who exhibited the lowest levels of knowledge, attitudes, and practices (scoring in the lowest 30th percentile). This sample size was determined to provide 95% confidence and 80% power to detect meaningful changes post‐intervention, assuming a moderate effect size. This approach aligns with methodologies used in prior interventional studies, where targeting high‐risk subgroups optimizes statistical power and clinical relevance [14, 15, 16]. Participants were selected from the initial pool of 400 to maximize measurable improvement in the highest‐risk subgroup. Pregnant women received an educational pamphlet containing food safety instructions. During a phone call, participants received a comprehensive explanation of this phase of the study and were asked to introduce a social network (such as Telegram or WhatsApp, …) through which the educational pamphlet would be distributed. Subsequently, each participant received the pamphlet via the chosen social network. After 2 weeks, a reminder call was made to ensure the pamphlet had been reviewed.

**Figure 1:**
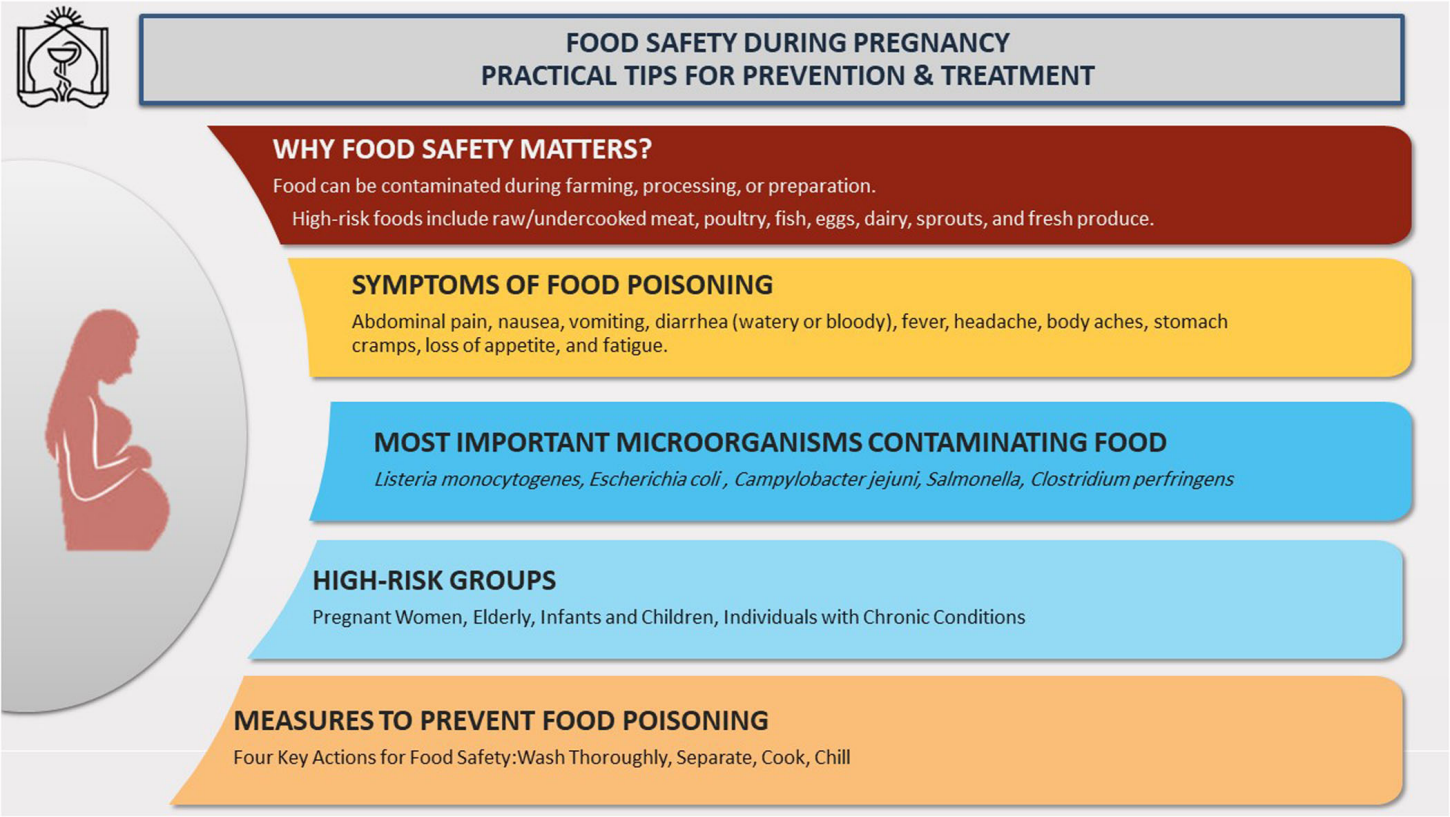
Summary of the key content of the educational pamphlet used in the intervention.

The pamphlet was developed based on recommendations from the WHO [17] and was written in fluent Farsi to ensure comprehensibility for laypeople (Supporting Information S1: File 1). Its validity was assessed by two food safety experts, ensuring the accuracy and appropriateness of the content. The pamphlet comprised seven sections presented in a question‐and‐answer format: “What are foodborne illnesses?,” “What are the symptoms of food poisoning?,” “What are the complications of food poisoning?,” “What are the most important microorganisms that contaminate food?,” “What measures are important to prevent food poisoning?,” “What should be considered when buying food?,” and “What should be considered when dining out (e.g., restaurants, catering, etc.)?” Figure 1 summarizes the key content of the educational pamphlet used in the intervention.

Two months after implementing the educational intervention with the tailored pamphlets, participants completed the same questionnaire to evaluate changes in their knowledge, attitudes, and practices.

### 2.5 Statistics

Statistics Data analysis was performed using SPSS statistical software (version 16, IBM Company, United States). All *p*‐values < 0.05 were considered statistically significant. Descriptive statistics were calculated, including means, standard deviations, and frequencies. Pre‐specified analyses were conducted to compare pre‐ and post‐intervention results using appropriate statistical tests, including independent *t*‐tests for comparing means between two independent groups, and paired *t*‐tests for comparing pre‐ and post‐intervention means within the same group.

### 2.6 Ethical Considerations

The study received ethical approval from the Ethics Committee at Mashhad University of Medical Sciences (IR.MUMS.MEDICAL.REC.1399.503). Before participation, all pregnant women were informed of the study's purpose, potential benefits, and the time commitment required. They were also informed of their right to withdraw from the study at any time without any impact on their healthcare services.

## 3 Result

In this study, 400 pregnant women completed the questionnaire in the first phase. 144 pregnant women with low knowledge, attitude, and practice entered the second phase of the study. The majority of participants were housewives (81.3%) between the ages of 16–31 (58.8%), with a college education (68.8%), and living most of their lives in a metropolis (82.3%). It was the first pregnancy of 30% of the pregnant women and 57.1% of them were in their third trimester (Table 1). Among the demographic data, only knowledge and education level had a significant relationship (*p* < 0.05). Other variables related to personal characteristics and pregnancy information of the two groups were similar before and after the intervention and there was no significant statistical difference using the *t*‐test (*p* > 0.05). The mean scores in the before and after educational intervention were 8.38 and 14.79 for overall knowledge, 40.73 and 44.39 for attitudes, and 42.91 and 47.75 for practices, respectively (Table 2). Based on the result, the mean scores of knowledge, attitude, and practice after the educational intervention had a statistically significant difference (*p* < 0.001).

**Table 1:** Demographic information of pregnant women participating in the study (*n* = 400).

Subject	(%)
Age (years)	
16–31 years	58.8
> 31 years	41.3
Educational levels	
Elementary school	2.5
Middle school	5
High school	23.8
College	68.8
Job‐status	
Housewife	81.3
Employee or Private Sector	18.8
Pregnancy Status	
First pregnancy	30.4
Second pregnancy	44.3
Third pregnancy	20.3
Fourth pregnancy	5.1
Pregnancy trimester	
First trimester	14.3
Second trimester	28.6
Third trimester	57.1

**Table 2:** Comparison of mean food safety knowledge, attitude, and practice of pregnant women in the intervention group (*n* = 144) before and after the educational intervention.

Variable	Measurement periods	Mean	Std. deviation	*p* value*
Knowledge	Before educational intervention	8.38	1.195	< 0.001
	After educational intervention	14.79	0.441	
Attitude	Before educational intervention	40.73	3.048	< 0.001
	After educational intervention	44.39	1.514	
Practice	Before educational intervention	42.91	1.917	< 0.001
	After educational intervention	47.75	0.626	

* *p* value from paired *t*‐tests comparing pre‐ and post‐intervention scores within the intervention group (*n* = 144).

## 4 Discussion

The present study was conducted to determine the impact of a food safety training program on the attitudes, awareness, and practice of pregnant women. The results showed that after implementing the educational program, the mean scores of knowledge, attitude, and practice increased compared to before the intervention. These results indicate the positive impact of the educational program on improving the knowledge, attitude, and practices related to food safety among pregnant women. This finding is consistent with similar studies conducted on pregnant women in various countries. Boyd et al. in the United States and Anderson in Scotland showed that nutritional education significantly improves nutritional knowledge and dietary behavior for pregnant women [18, 19]. Shabbier et al. and Shakeri conducted studies in Iran that showed nutritional education programs for pregnant women led to improved nutritional behaviors in the intervention group compared to the control group [20, 21]. Fallah et al. demonstrated notable enhancements in the level of awareness among pregnant women who participated in a minimum of two educational sessions focusing on healthy nutrition. The awareness level increased significantly from 3% before the intervention to 31% following the nutritional education program (*p* < 0.001) [22]. Verbeke identified that providing education on nutrition and food consumption has the potential to address safety concerns within the population [23]. Also, in the study of Widga and Lewis, after training, a significant improvement was observed in the consumption of fruits and vegetables, folates, energy, calcium, and vitamins in pregnant women [24]. However, the results of some studies show the lack of positive effects of educational interventions on improving nutritional behavior [25, 26, 27]. The reason for the difference in the results of these studies may be attributed to differences in the methods of delivering training and educational interventions [28]. Studies have shown that the use of various educational methods, in terms of the type and method of providing educational interventions, can have a profound effect on the outcomes [28]. In this study, an educational pamphlet was used to present the content, which had positive results. The lack of significant positive impact of nutritional and food safety education can be related to other factors such as lifestyle [13, 29], community beliefs [18, 30], economic issues [28, 31], and access to food [13, 30].

### 4.1 Limitations

This study faces several limitations. The majority of participants were college‐educated, urban‐dwelling housewives, which does not adequately represent the diverse socioeconomic backgrounds and living conditions of the broader pregnant population. This lack of diversity may limit the applicability of our results to different demographic groups, particularly those who may face varying levels of food insecurity, nutritional challenges, and other health disparities. Moreover, the decision to focus exclusively on “healthy” pregnant individuals introduces an additional layer of bias. Individuals with health issues are more likely to encounter food insecurity and malnutrition, which can further complicate their nutritional status and pregnancy outcomes. Consequently, the findings of this study may not fully capture the complexities associated with nutrition among all pregnant individuals, particularly those who are at higher risk. Additionally, the validation of the measurement instrument used in this study was conducted with “experts” rather than the population for which the instrument was ultimately intended. The perspectives and experiences of the actual population may differ from those of the experts. We suggest that future research should aim to include a more diverse sample, encompassing a broader range of educational backgrounds and health statuses. This could provide a more comprehensive understanding of food safety knowledge and practices among pregnant women in various socioeconomic contexts.

## 5 Conclusions

This study revealed that knowledge, attitude, and practice change through educational courses were effective in improving the food safety status of pregnant women. Increasing the level of knowledge, attitude, and practice of pregnant women about food safety using educational pamphlets is a low‐cost and suitable intervention to improve the food safety status of pregnant women. This intervention has the potential to be replicated in a broader population through enhanced collaboration among government agencies, nongovernmental organizations, and providers of maternal and child health services.

## Author Contributions

**Fateme Asadi Touranlou:** conceptualization, writing – original draft. **Mohammad Hashemi:** visualization, writing – review and editing. **Mahsa Neghabi:** data curation, investigation. **Shiva Adibi:** data curation, investigation. **Asma Afshari:** supervision, writing – review and editing, visualization, project administration.

## Conflicts of Interest

The authors declare no conflicts of interest.

## Transparency Statement

The lead author Asma Afshari affirms that this manuscript is an honest, accurate, and transparent account of the study being reported; that no important aspects of the study have been omitted; and that any discrepancies from the study as planned (and, if relevant, registered) have been explained.

## Supporting information

Supplementary File 1.

## Data Availability

The authors confirm that the data supporting the findings of this study are available within the article and its supplementary information.
