# Supplementary material for: Evaluation of the Effect of Educational Courses Containing Food Safety Instructions on the Level of Knowledge, Attitude, and Practice of Pregnant Women: A Cross‐Sectional Study
Source: Health Sci Rep. 2025 Oct 13;8(10):e71337. doi: 10.1002/hsr2.71337 (PMC12516226; doi:10.1002/hsr2.71337)
Supplement: Supplementary file 1 [file HSR2-8-e71337-s001.docx]

**Food Safety During Pregnancy: Practical Tips for Prevention and Treatment**

**Introduction**

Food can become contaminated at various stages, including farming, harvesting, processing, packaging, transportation, storage, and preparation. High-risk foods include raw or undercooked red meat, poultry, fish, eggs, dairy products, sprouts, and fresh fruits and vegetables. Contamination often occurs due to the transfer of harmful bacteria between surfaces, especially with raw and ready-to-eat foods like salads, which are not cooked to eliminate pathogens. Proper food preparation and specific precautions can significantly reduce the risk of foodborne illnesses.

**1. What are foodborne illnesses?**

**Definitions**

- **Foodborne Illnesses**: Diseases caused by consuming food or drinks contaminated with microorganisms (bacteria, parasites, viruses) or chemical contaminants, also known as food poisoning.
- **Food Safety**: Conditions and practices that maintain food quality to prevent contamination and foodborne illnesses.

**High-Risk Groups**

- **Elderly**: Reduced immune system efficiency with age.
- **Pregnant Women**: Metabolic and circulatory changes increase susceptibility, with potentially severe reactions. In rare cases, the fetus may also be affected.
- **Infants and Children**: Immature immune systems.
- **Individuals with Chronic Conditions**: Diseases like diabetes, liver disease, AIDS, or those undergoing chemotherapy/radiation weaken immune responses.

**Severe Foodborne Illnesses**

- **Listeria monocytogenes**: Particularly dangerous for pregnant women, potentially causing miscarriage, stillbirth, premature delivery, or severe newborn infections. Survivors may face long-term neurological or developmental issues.
- **Escherichia coli (E. coli)**: Certain strains can cause hemolytic uremic syndrome, leading to kidney failure, especially in the elderly, children under 5, and those with weakened immune systems.

**2. What are the symptoms of food poisoning?**

**Symptoms of Food Poisoning**

Symptoms vary depending on the contaminant but often include:

- Abdominal pain, nausea, vomiting, diarrhea (watery or bloody), fever, headache, body aches, stomach cramps, loss of appetite, and fatigue.
- Symptoms may appear hours to days after consuming contaminated food and typically last 1–10 days.
- Severity depends on the type and amount of contaminant, as well as the individual’s age and health.

**Treatment for Food Poisoning**

- **Rest the Stomach**: Avoid eating/drinking for a few hours.
- **Hydrate**: Sip small amounts of water, suck on ice chips, or drink caffeine-free clear sodas. Adults should consume 8–16 cups of fluids daily in small, frequent sips.
- **Monitor Urine**: Normal volume and clear color indicate adequate hydration.
- **Resume Eating Gradually**: Start with simple, easily digestible foods. Stop if nausea returns.
- **Avoid Certain Foods**: Refrain from dairy, caffeine, fatty, or spicy foods until recovered.
- **Rest**: Food poisoning and dehydration can cause weakness and fatigue.

**When to Seek Medical Attention**

Consult a doctor immediately if you experience:

- Vomiting lasting more than 2 days
- Blood in vomit or stool
- Severe diarrhea for more than 3 days
- Severe abdominal pain or cramps
- Oral temperature above 38.6°C (101.5°F)
- Signs of dehydration (extreme thirst, dry mouth, reduced urination, severe weakness, dizziness) Provide your doctor with detailed symptoms, foods consumed, where they were eaten, and when symptoms began. Diagnosis is often based on medical history, symptoms, and consumed foods.

**3. What are the complications of food poisoning?**

**Complications**

The most serious complication is **dehydration** (severe loss of body water and electrolytes). Healthy adults who replace fluids lost through diarrhea and vomiting typically avoid issues. However, high-risk groups (e.g., pregnant women, elderly) are more prone to severe dehydration, which may require hospitalization for intravenous fluids and electrolytes. Early treatment, especially for listeriosis, can prevent transmission to the fetus and reduce severity.

**4. What are the most important microorganisms that contaminate food?**

**Common Foodborne Pathogens**

| Pathogen | Contaminated Foods | Onset | Symptoms |
| --- | --- | --- | --- |
| *Campylobacter jejuni* | Contaminated water, unpasteurized milk, raw/undercooked meat | 2–5 days | Fever, headache, muscle pain, followed by diarrhea (sometimes bloody), abdominal pain, nausea (7–10 days). Can spread to blood, potentially fatal. |
| *Clostridium perfringens* | Food left at room temperature, meats, broths | 8–22 hours | Abdominal cramps, diarrhea (24 hours, up to 1–2 weeks in elderly). Rarely fatal. |
| *Listeria monocytogenes* | Deli meats, unpasteurized dairy, raw produce, smoked seafood, salads | 9–48 hours (up to 3 weeks) | Fever, chills, headache, back pain, upset stomach, abdominal pain, diarrhea. Severe in high-risk groups; can cause miscarriage, stillbirth, or newborn death. |
| *E. coli O157:H7* | Contaminated meat, undercooked hamburger, unpasteurized juice/milk, raw produce | 1–8 days | Severe (often bloody) diarrhea, muscle cramps, vomiting, mild/no fever (5–10 days). Can cause hemolytic uremic syndrome (kidney failure). |
| *Norovirus* | Contaminated water/food, salads, sandwiches, cakes, ice cream | 12–48 hours | Nausea, vomiting, abdominal pain, fever, muscle aches, headache (1–2 days). Diarrhea common in adults, vomiting in children. |
| *Salmonella* | Raw/undercooked meat, poultry, eggs, unpasteurized dairy, raw produce | 1–3 days | Stomach pain, diarrhea, nausea, chills, fever, headache (4–7 days). Can spread to blood, potentially fatal if untreated. |
| *Vibrio vulnificus* | Raw/undercooked seafood | 1–7 days | Diarrhea, abdominal pain, vomiting (2–8 days). Can cause bloodstream infection, potentially fatal in immunocompromised. |
| *Toxoplasma gondii* | Cat feces, raw/undercooked meat | 5–23 days | Flu-like illness (months in severe cases). Can cause miscarriage in pregnant women. |
| *Clostridium botulinum* | Unpasteurized whey, improperly canned foods, honey | 12–24 hours | Blurred/double vision, drooping eyelids, motor impairment, speech/swallowing issues, sore throat. |
| *Cryptosporidium* | Contaminated water, raw/undercooked food, soil | 7–10 days | Watery diarrhea, dehydration, weight loss, stomach pain, fever, nausea, vomiting (2–14 days, longer in immunocompromised). |
| *Giardia lamblia* | Raw/ready-to-eat food, contaminated water | 1–2 weeks | Spread via contaminated food handler. |
| *Hepatitis A* | Raw/ready-to-eat food, contaminated seafood | 8 days | Spread via contaminated food handler. |
| *Rotavirus* | Raw/ready-to-eat food | 1–3 days | Spread via contaminated food handler. |
| *Shigella* | Raw/ready-to-eat food | 24–48 hours | Spread via contaminated food handler. |
| *Staphylococcus aureus* | Meats, salads, creamy sauces, pastries | 1–6 hours | Spread via hand contact, coughing, sneezing. |

**5. What measures are important to prevent food poisoning?**

**Four Key Actions for Food Safety**

**1. Wash Thoroughly**

Microorganisms can spread in the kitchen, contaminating surfaces, utensils, and cutting boards. Follow these steps:

- **Hand Washing**: Wash hands with warm water and soap before and after handling food (especially raw meat), using the bathroom, changing diapers, or touching pets.
- **Clean Utensils and Surfaces**: Wash cutting boards, dishes, and surfaces with hot water and detergent immediately after use.
- **Clean Refrigerator**: Regularly clean inside and outside to remove food drippings.
- **Vegetable Washing**: Rinse vegetables to remove dirt, disinfect with approved products (per instructions), and rinse again.
- **Avoid Contamination**: Do not prepare food with open cuts or wounds unless wearing gloves. Avoid touching wounds on other body parts during food prep.

**2. Separate**

Prevent cross-contamination by keeping raw and cooked foods apart:

- Separate raw meat, poultry, seafood, and their juices from ready-to-eat foods during shopping, preparation, and storage.
- Use separate cutting boards for raw meats and produce.
- Store cooked or ready-to-eat foods in clean containers, not in containers previously used for raw meat.
- Cover foods in the refrigerator to prevent drips.

**3. Cook**

Cook foods to safe internal temperatures to kill harmful microorganisms:

- Most foods require 63–74°C (145–165°F). Use a clean food thermometer to check.
- Cook eggs until yolks and whites are firm. Avoid raw or undercooked egg dishes.
- Avoid raw or undercooked meat, poultry, fish, and sprouts.
- Use pasteurized dairy and juices.
- Cook meats to: red meat (70–80°C), whole poultry (82°C), fish/seafood (63°C), eggs (71°C).
- Shrimp and lobster should be cooked until opaque and red; fish should be non-translucent.
- Reheat leftovers (e.g., soups, stews) to boiling or 74°C. Stir and rotate food in microwaves for even heating.
- Do not consume marinades used for raw meat unless boiled.
- Boil canned foods for 20 minutes before consumption.

**4. Chill**

Proper refrigeration prevents bacterial growth:

- Set refrigerators to 4°C (40°F) or below and freezers to -18°C (0°F) or below.
- Refrigerate perishable foods within 2 hours of purchase or preparation (1 hour if air temperature exceeds 30°C/86°F).
- Freeze foods not consumed within 2 days.
- Thaw frozen foods safely in the refrigerator, microwave (on defrost setting), or cold running water, then cook immediately.
- Do not overfill refrigerators/freezers to allow cold air circulation.
- Keep marinating foods in the refrigerator.
- Discard food left at room temperature for too long, as cooking may not eliminate toxins.
- During power outages, keep refrigerator/freezer doors closed (refrigerators stay cold for ~4 hours, full freezers for ~48 hours).

**Additional Safety Tips**

- Spoiled food may look and smell normal but still be unsafe.
- Avoid storing food in the 4–60°C (40–140°F) danger zone where bacteria thrive.
- Discard expired or suspicious foods without tasting.
- Mold indicates spoilage; discard moldy food, even from the refrigerator.
- Follow storage instructions on packaging.
- Food poisoning can be severe or fatal in high-risk groups.
- Avoid drinking from rivers or springs.
- Use proper, hygienic methods for home canning to prevent botulism.
- Do not give honey to children under 1 year.
- Avoid wild mushrooms.
- Warn others if you suspect food you ate caused illness.
- Store potatoes and onions in cool, dry places, not under sinks.
- Keep food away from chemicals or cleaning products.

**Storage Guidelines**

| Food Item | Refrigerator (4°C) | Freezer (-18°C) |
| --- | --- | --- |
| Fresh eggs in shell | 4–5 weeks | Do not freeze |
| Raw egg yolk/white | 2–4 days | 1 year |
| Hard-boiled eggs | 1 week | Do not freeze |
| Mayonnaise (opened) | 2 months | Do not freeze |
| Soup/stew (with vegetables/meat) | 3–4 days | 2–3 months |
| Sausage | 1–2 days | 1–2 months |
| Meat/organs (e.g., liver) | 1–2 days | 3–4 months |
| Cooked meat leftovers | 3–4 days | 2–3 months |
| Broth | 1–2 days | 2–3 months |
| Whole fresh chicken | 1–2 days | 1 year |
| Cut fresh chicken | 1–2 days | 9 months |
| Fried chicken leftovers | 3–4 days | 4 months |
| Cooked chicken | 3–4 days | 4–6 months |
| Chicken nuggets | 1–2 days | 1–3 months |
| Lean fish | 1–2 days | 6 months |
| Fatty fish | 1–2 days | 2–3 months |
| Cooked fish | 3–4 days | 4–6 months |

**Choosing Safer Foods**

| Food Type | Higher Risk | Lower Risk |
| --- | --- | --- |
| Meat/Poultry | Raw or undercooked | Cooked to safe internal temperature |
| Seafood | Raw, undercooked, or refrigerated smoked | Canned or cooked to 63°C; reheated to 74°C |
| Milk | Unpasteurized | Pasteurized |
| Eggs | Raw, undercooked, or dishes with raw eggs | Fully cooked (firm yolk and white) |
| Sprouts | Raw | Cooked |
| Vegetables | Unwashed fresh | Thoroughly washed fresh |
| Cheese | Made from unpasteurized milk | Made from pasteurized milk |
| Hot dogs/Deli meats | Cold or not reheated properly | Reheated to 74°C (steaming) |

**6. What should be considered when buying food?**

**Safe Food Shopping**

- Choose high-quality, safe ingredients.
- Avoid foods with unusual odors or tastes.
- Place perishable items (meat, dairy, eggs) in your cart last and store separately.
- Wrap raw meat in plastic bags before placing in the cart.
- Refrigerate perishables within 1 hour of purchase.
- Check production and expiration dates; avoid expired or suspicious-looking products.
- Avoid cracked eggs or fruits with broken skins.
- Choose pasteurized dairy and juices from refrigerated sections.
- Check labels for manufacturing licenses and factory details.
- Avoid damaged cans (swollen, leaking, dented, rusted, or sticky). Boil canned foods for 20 minutes before use.

**7. What should be considered when dining out (e.g., restaurants, catering, etc.)?**

**Eating Out**

- Choose clean, hygienic restaurants.
- Order fully cooked foods; avoid raw or undercooked meat or eggs.
- Ensure food is served hot. Avoid eating if it’s not.
- Discard food left out for over 2 hours.
- Refrigerate takeout within 2 hours or keep hot (≥74°C) to prevent bacterial growth.
- Transport food home quickly to refrigerate, as car interiors can get very hot.

**Traveling**

- Consume only hot, freshly cooked foods.
- Boil drinking water and avoid raw vegetables or unpeeled fruits.
- Keep cold foods at ≤4°C with ice packs and hot foods at ≥74°C in insulated containers.
